# MS-20 enhances the gut microbiota-associated antitumor effects of anti-PD1 antibody

**DOI:** 10.1080/19490976.2024.2380061

**Published:** 2024-07-30

**Authors:** Pei-Jung Lee, Chien-Min Hung, Ai-Jen Yang, Cheng-Yu Hou, Hung-Wen Chou, Yi-Chung Chang, Wen-Cheng Chu, Wen-Yen Huang, Wen-Chih Kuo, Chia-Chun Yang, Kuo-I Lin, Kuo-Hsuan Hung, Li-Chun Chang, Kang-Yun Lee, Han-Pin Kuo, Kung-Ming Lu, Hsin-Chih Lai, Ming-Liang Kuo, Wan-Jiun Chen

**Affiliations:** a Pharmaceutical Research & Development, Microbio Co, Ltd, Taipei, Taiwan; b Animal Center for Drug Screening, Oneness Biotech Co, Ltd, Taipei, Taiwan; c Nucleic Acid Drug Division, Microbio (Shanghai) Biotech Company, Shanghai, China; d Genomics Research Center, Academia Sinica, Taipei, Taiwan; e Division of Gastroenterology, Department of Internal Medicine, National Taiwan University Hospital, Taipei, Taiwan; f Division of Pulmonary Medicine, Department of Internal Medicine, Shuang Ho Hospital, Taipei Medical University, New Taipei City, Taiwan; g Pulmonary Medicine Research Center, Taipei Medical University, Taipei, Taiwan; h Department of Thoracic Medicine, Taipei Medical University Hospital, Taipei, Taiwan; i General manager’s office, Microbio Co., Ltd., Taipei, Taiwan; j General manager’s office, Revivebio Co, Ltd, Taipei, Taiwan

**Keywords:** Gut microbiota, cancer immunotherapy, colorectal cancer

## Abstract

Cancer immunotherapy has been regarded as a promising strategy for cancer therapy by blocking immune checkpoints and evoking immunity to fight cancer, but its efficacy seems to be heterogeneous among patients. Manipulating the gut microbiota is a potential strategy for enhancing the efficacy of immunotherapy. Here, we report that MS-20, also known as “Symbiota®”, a postbiotic that comprises abundant microbial metabolites generated from a soybean-based medium fermented with multiple strains of probiotics and yeast, inhibited colon and lung cancer growth in combination with an anti-programmed cell death 1 (PD1) antibody in xenograft mouse models. Mechanistically, MS-20 remodeled the immunological tumor microenvironment by increasing effector CD8^+^ T cells and downregulating PD1 expression, which were mediated by the gut microbiota. Fecal microbiota transplantation (FMT) from mice receiving MS-20 treatment to recipient mice increased CD8^+^ T-cell infiltration into the tumor microenvironment and significantly improved antitumor activity when combined with anti-PD1 therapy. Notably, the abundance of *Ruminococcus bromii*, which increased following MS-20 treatment, was positively associated with a reduced tumor burden and CD8^+^ T-cell infiltration *in vivo*. Furthermore, an *ex vivo* study revealed that MS-20 could alter the composition of the microbiota in cancer patients, resulting in distinct metabolic pathways associated with favorable responses to immunotherapy. Overall, MS-20 could act as a promising adjuvant agent for enhancing the efficacy of immune checkpoint-mediated antitumor therapy.

## Introduction

Despite breakthroughs in immunotherapies for cancer, nearly 70% of patients are regarded as nonresponders.^1,2^ Certain species and strains of the gut microbiota have been proposed to regulate the immune system and are considered one of the strategies that can be used to improve the response to immunotherapy.^3^ For instance, a defined commensal consortium was reported to elicit intestinal CD8^+^ T cells and anticancer immunity in combination with anti-PD1 antibody therapy.^4^ Oral administration of *Bifidobacterium* showed antitumor activity and promoted the entry of CD8^+^ T cells into the tumor microenvironment when administered in combination with an anti-PD-L1 antibody.^5^ The gut microbiota profiles of immunotherapy responders and nonresponders were distinct. In melanoma patients, the abundance of bacteria such as Bacteroidales increased in nonresponders and was negatively correlated with intratumoral CD3^+^ and CD8^+^ immune cell levels. In contrast, Clostridiales, Ruminococcus and *Faecalibacterium* were enriched in responders and were positively correlated with CD3^+^ and CD8^+^ immune cell levels.^6^ In addition, specific bacteria, such as *Coprobacillus cateniformis*, might inhibit tumor growth by downregulating the PD-L2-RGMb pathway when combined with anti-PD-L1 treatment.^7^ Recently, *Enterocloster* spp. were found to promote the entry of immunosuppressive enterotropic α4β7^+^CD4^+^ regulatory T 17 cells into the tumor microenvironment through the downregulation of mucosal vascular addressin cell adhesion molecule 1 (MAdCAM-1) expression in high endothelial venules.^8^ Taken together, these studies indicated that the gut microbiota plays a vital role in regulating immune checkpoint marker expression and associated treatment efficacy.

Previous studies have shown that fecal microbiota transplantation (FMT) from immunotherapy responders plus anti-PD1 blockade increases the efficacy of tumor reduction in mouse models. A greater density of CD8^+^ T cells in the tumor microenvironment is observed in mice that received fecal transplantation from immunotherapy responders.^9,10^ Several clinical trials have also been undertaken to enhance the response to immunotherapy by modulating the gut microbiota.^11^ In a single-center, double-arm, open-label phase 1 study, patients with advanced renal cell carcinoma who received immunotherapies plus live bacterial products (CBM588 containing *Clostridium butyricum*) had longer progression-free survival (PFS) than did those who received immunotherapies alone.^12^ By introducing feces from patients who responded to immunotherapy, two studies reported that the clinical response rates were 30% (3 of 10) and 20% (3 of 15) in patients with refractory melanoma who were reintroduced to anti-PD1 therapy,^13,14^ which were higher than that in patients who were retreated with anti-PD1 therapy alone (5 out of 34, 14.7%).^15^ For treatment-naïve patients with advanced melanoma who received immunotherapy in combination with FMT from healthy donors, the objective response rate was 65% (13 of 20),^16^ which was superior to that achieved with immunotherapy alone (42%-45%).^17,18^ Collectively, these data suggested that modulating the gut microbiota is a promising therapeutic strategy to improve the clinical response to cancer immunotherapy.

MS-20 is a fermentation product generated by a consortium of *Lactobacillus* and yeast that are subsequently grown in soybean-based media, after which the secondary metabolites are extracted and concentrated.^19^ A previous study showed that MS-20 (described as the fermented soy milk product, FSP) induced the apoptosis of breast cancer cells and inhibited tumor growth in a mouse xenograft model without severe side effects.^20^ MS-20 (or soybean fermentation broth [SFB]) also increased the proliferation of T cells, enhanced the production of the Th1-related cytokine IL-2, inhibited the production of the Th2-related cytokine IL4, and promoted NK cell activity *in vivo*.^21^ The immune-enhancing effects of MS-20 were also observed in human clinical trials. The reduction in NK cell activity during chemotherapy was enhanced in the presence of MS-20.^19^ Thus, the fermented soybean extract MS-20 exerted immunomodulatory effects. As the manipulation of the gut microbiota has therapeutic potential in cancer immunotherapy, in this study, we sought to determine whether MS-20 administration may enhance the efficacy of immunotherapy.

## Materials and methods

### Xenograft model

#### Prevention model

All animal studies were approved by the Animal Center for Drug Screening, Oneness Biotech Co., Ltd. A total of 2 × 10^5^ viable CT-26 cells were subcutaneously injected into BALB/c mice (LASCO, Taipei, Taiwan). Next, the mice were randomized and orally administered vehicle control (sterile water) or 1%, 5% or 15% MS-20 (10 mL/kg; Microbio Co., Ltd., Taipei, Taiwan) daily. Mice were intraperitoneally administered anti-PD1 antibody (10 mg/kg; BioXcell; BE0146, clone: RMP1–14) or the isotype control (BioXcell; BE0089) on days 6, 8, 10 and 12.

#### Treatment model

A total of 2 × 10^5^ viable CT-26 cells were subcutaneously injected into BALB/c mice. Three days after implantation, the tumor-bearing mice were randomized into groups and dosed daily with vehicle control or 1%, 5% or 15% MS-20 by oral gavage. Mice were treated with 10 mg/kg anti-PD1 antibody or the isotype control by intraperitoneal injection on days 3, 5, 7, 9 and 11. For the CD8 neutralization experiment, 10 mg/kg anti-CD8 antibody (BioXcell; BE0061, clone: 2.43) or the isotype control (BioXcell; BE0090) was intraperitoneally injected into the mice on the same day as the anti-PD1 antibody.

For the lung cancer model, 10^6^ viable LL/2 cells were subcutaneously injected into C57BL/6 mice (LASCO, Taipei, Taiwan). Four days after implantation, the tumor-bearing mice were randomized into groups and orally gavaged with 15% MS-20 (10 mL/kg) or vehicle control daily. Mice were treated with 10 mg/kg anti-PD1 antibody by intraperitoneal injection on days 0, 2, 4, 6 and 8.

#### FMT model

Ampicillin (1 g/L, Bioshop, AMP201.5), neomycin (1 g/L, Bioshop, NEO201.25), metronidazole (1 g/L, Sigma, M1547) and vancomycin (0.5 g/L, Sigma, V2002) were dissolved in sterile water. Drinking water containing antibiotics was freshly prepared and changed twice a week. After the antibiotic treatment, 2 × 10^5^ viable CT-26 cells were subcutaneously injected into BALB/c mice. On day 4 postimplantation, the tumor-bearing mice were randomized into groups and orally gavaged with FMT (100 μL/mouse) daily. Mice were administered 5 mg/kg anti-PD1 antibody by intraperitoneal injection on days 6, 8, 10, and 12.

### FMT stool collection

Feces from donor mice (2–3 fecal pellets from each mouse) were collected daily and pooled in sterile tubes. One hundred milligrams of feces was resuspended in 1 ml of PBS and homogenized to obtain a liquid slurry. Then, the feces were centrifuged at 2000 × g at 4°C for 1 min. The bacteria-enriched supernatants were collected and centrifuged for 5 min at 15,000 × g and 4°C. Bacterial pellets were resuspended in 1 ml of PBS supplemented with 20% (v/v) glycerol and stored at −80°C.

### Flow cytometry

Tumor-infiltrating immune cells were isolated with a tumor dissociation kit (Miltenyi Biotec, 130-096-730) according to the manufacturer’s instructions. The cells were stained with the live stain (BD 564,996) for 15 min at room temperature. After centrifugation at 300 × g for 5 min, the cells were washed with staining buffer and incubated with Fc-blocking buffer (BD 553,142) to prevent nonspecific binding for 10 min on ice. Next, the cells were incubated with CD45-BV510 (BD 567,800), CD3-PerCP-Cy5.5 (BD 566,495), CD4-FITC (BD 553,046), CD8-APC-Cy7 (BD 557,654), CD25-PE-Cy7 (BD 552,880), CD11c-R718 (BD 565,872), MHC-II-BV786 (BD 742,894), CD62L-BV650 (BD 564,108), CD44-APC (BD 559,250) and CD279-BV421 (BD 562,584) antibodies in FACS buffer at 4°C for 30 min. The cells were then washed twice with staining buffer. Foxp3 was stained with a PerFix-nc kit (Beckman Coulter, B31167) according to the manufacturer’s instructions. Briefly, the cells were suspended in 50 μl of PBS, 25 μl of fixative reagent was added, and the mixture was mixed gently. After an incubation for 15 min at room temperature in the dark, the cells were stained with 200 μl of permeabilization reagent containing an anti-Foxp3-PE antibody (BD 560,408). After an incubation for 60 min at room temperature in the dark, the cells were washed with PBS. Then, 250 μl of 1×Reagent 3 was added, and the mixture was incubated for 5 min at room temperature. The cells were then washed with PBS, resuspended in 200 μl of 1×Reagent 3 and analyzed using flow cytometry on a CytoFLEX instrument (Beckman).

### Ex vivo culture of fecal microbiota

Fecal stock samples were aliquoted in tubes and stored at −80°C. For each experiment, frozen stock tubes were thawed in a 37°C water bath. The feces were diluted to 5 × 10^7^ CFU/ml with MiPro medium and incubated with the vehicle control (H_2_O) or MS-20 (0.625%, 1.25% and 2.5%) for 24 h under anaerobic conditions. Bacterial pellets were collected by centrifugation, and bacterial gDNA was extracted with a Presto^TM^ gDNA Bacteria Advanced Kit according to the manufacturer’s instructions. The samples were procured and utilized according to approved IRB protocols for research on human subjects (TMUH N202012003, NTUH 202211061RIFD). Written informed consent was obtained from all patients. Patient characteristics are listed in Table S1.

### IHC staining

Tumor tissues were fixed with 10% paraformaldehyde. The tumor sections were incubated at 65°C for 30 min and deparaffinized with EZ buffer. The sections were then incubated in antigen retrieval buffer for 90 min and treated with 3% H_2_O_2_ for 4 min. Next, the sections were incubated with an anti-CD8 antibody (Synaptic Systems; HS-361003) or anti-PD1 antibody (R&D; AF1021) at 37°C for 2 h and then incubated with a secondary antibody (N-Histofine simple stain mouse MAX PO; 414341F for CD8; 414161F for PD1) at room temperature for 40 min. Staining was visualized using diaminobenzidine (Thermo), followed by counterstaining with hematoxylin (Sigma) and bluing. HALO software (Indica Labs) was used to quantify CD8^+^ and PD1^+^ cells after whole-slide scanning using an Aperio AT2 instrument (Leica, Wetzlar, Germany).

### Real-time RT‒PCR

The expression levels of related genes were determined by performing real-time reverse transcription PCR (RT-qPCR) using an ABI Prism 7900 sequencer (Applied Biosystems, Foster City, CA, USA). TBP was used as an internal control. The expression levels were normalized to those of the internal control and defined as ΔCT=[CTtarget-CTinternal control]. The relative expression ratio was calculated as the fold change relative to the control (2^−△△CT^).

### Immunofluorescence staining

The tumors were frozen in liquid nitrogen, embedded in OCT, and stored at −80°C. The blocks were sectioned at a thickness of 5 µm and placed on slides. The sections were incubated with ice-cold blocking solution (PBS containing 5% goat serum) for 30 min, the primary antibody (anti-CD8 antibody; Abcam; ab22378; 1:200) was diluted in blocking solution, and the sample was incubated overnight at 4°C. After three washes with PBS, the tissues were incubated with a Texas red-conjugated secondary antibody (Abcam; ab7095; 1:500; goat anti-rat IgG (H+L) conjugate) for 1 h. The nuclei were stained with EverBrite mounting medium with DAPI (Biotium). The stained cells were examined using a confocal laser scanning microscope (C1 si, Nikon, Japan) with MetaXpress (Molecular Devices, Sunnyvale, CA, USA).

### Metagenomic sequencing and analysis

Whole-genome shotgun libraries were prepared using the TruSeq DNA PCR-Free Sample Preparation Kit (Illumina). Three hundred bp at each end of the libraries were sequenced on a MiSeq instrument with the MiSeq Reagent Kit v3 (600 cycles; Illumina). Data processing and the microbial community analysis were conducted using MetaPhlAn4 with the database version mpa_vOct22_CHOCOPhlAnSGB_202212. This tool allowed for the identification and quantification of microbial entities present in the samples at a high resolution, thereby leveraging its extensive database of clade-specific marker genes. The shotgun sequencing approach not only complemented the 16S rRNA gene analysis for a more detailed microbial community profile but also facilitated the exploration of microbial functional capabilities beyond the taxonomic composition.

### Microbial community analyses

gDNA was extracted from fecal samples using the Mag-Attract Power Microbiome DNA/RNA KF Kit (Qiagen). The V3-V4 regions of the 16S rRNA gene were amplified using the primers listed in the supplementary table and purified and sequenced using the MiSeq Illumina pyrosequencing platform according to the manufacturer’s instructions. The microbial community composition was analyzed using both 16S rRNA gene sequencing and shotgun metagenomic sequencing approaches. For the 16S rRNA gene analysis, we utilized the QIIME 2 (2021.11) platform. Sequence denoising was performed using deblurring to generate amplicon sequence variants (ASVs), which served as the basis for further analysis. Taxonomic assignment of ASVs was conducted using the Taxonomy Blast tool against the Silva database (release 138), which enables precise microbial identification at various taxonomic levels. In the analysis of β-diversity to assess the differences in microbial community composition among groups, we employed the pairwise.adonis function from the pairwiseAdonis package in R. This method utilizes a permutational multivariate analysis of variance (PERMANOVA) approach based on a permutation testing framework to evaluate the significance of the differences in community composition captured by our β-diversity metrics. Our β-diversity analysis was based on Bray‒Curtis dissimilarity, which was calculated from the microbial abundance data. PERMANOVA was conducted with 999 permutations to assess the statistical significance of the observed differences in microbial communities across the studied groups.

### Transcriptomic analysis

Colon tissues were homogenized using a TissueLyser (Qiagen, Hilden, Germany) with homogenization beads. RNA was isolated using GENEzol TriRNA Pure kits (Geneaid Biotech, New Taipei City, Taiwan). The microarray experiments and data analysis were performed by Welgene Biotech (Taipei, Taiwan) using Agilent Oligo Chips. The differentially expressed genes were subjected to Gene Ontology (GO) and KEGG pathway analyses to identify the significantly enriched pathways.

### Co-occurrence networks

The phyloseq object in the Phyloseq R package, version 1.40.0, was applied throughout the analysis.^22^ The estimation of the co-occurrence network was performed utilizing the SPIEC-EASI (SParse InversE Covariance Estimation for Ecological Association Inference) R package, version 1.1.2,^23^ where the neighborhood selection model with specific parameters (λ.min.ratio = 5e-2, nλ = 15, and 50 replicates) was used. Subsequent visualization of the network was achieved through the ggnet2 function of the ggnet R package, version 0.1.0,^24^ which is an extension of ggplot2.

### Statistics

The quantitative *in vitro* and *in vivo* data are presented as the mean±s.d., unless noted otherwise. Statistical significance was assessed using the two-tailed Student’s t test for pairwise comparisons of groups. The differences were assessed using Student’s t test. *p ≤ 0.05, **p ≤ 0.01 and ***p ≤ 0.001.

## Results

### MS-20 in combination with an anti-PD1 antibody inhibited tumor growth in colon and lung cancer models in vivo

**Figure 1.**
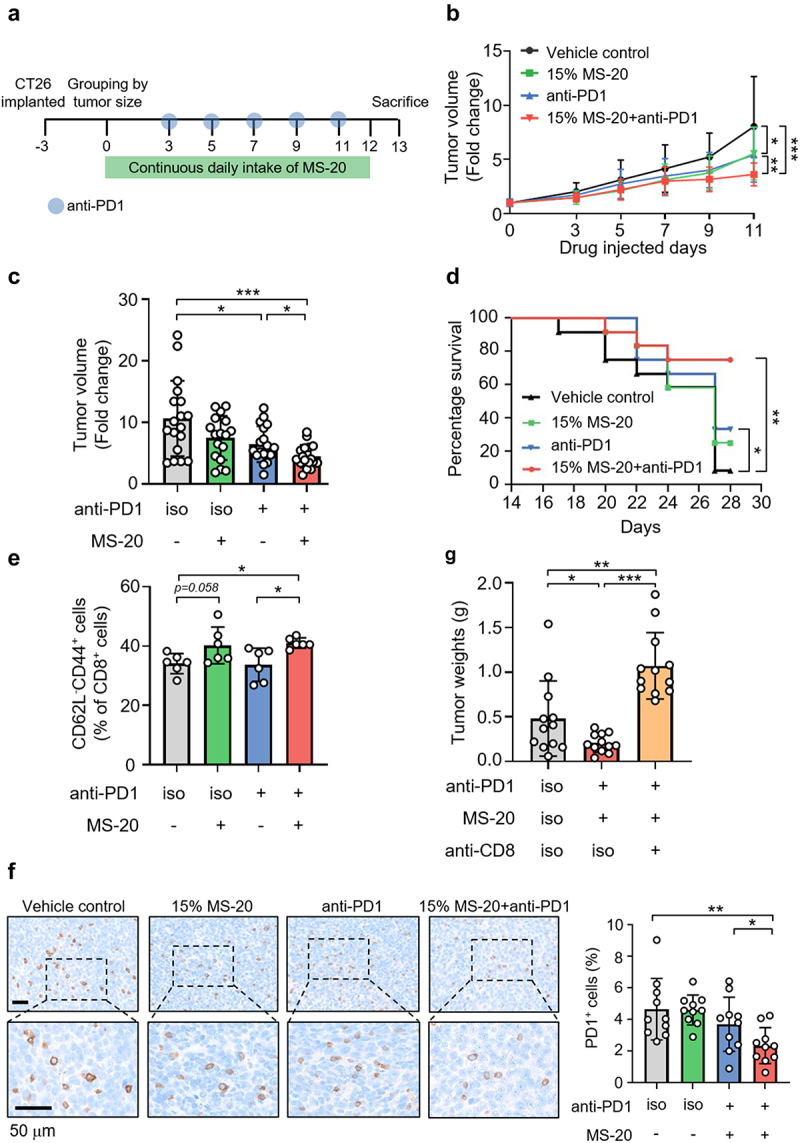
MS-20 plus an anti-PD1 antibody inhibited colon cancer growth in vivo.

**Note**: CT26 cells (2 × 10^5^) were subcutaneously injected into BALB/c mice. After tumor growth, the mice were randomly assigned to the vehicle control, 15% MS-20, anti-PD1 or 15% MS-20 plus anti-PD1 groups (a). Tumor growth was recorded (*n* = 17-18/group). The tumor volume was normalized to that on day 0, and the data represent the combination of two independent replicates (b). The tumor volume on the day of sacrifice was normalized to that on day 0, and the data represent the combination of two independent replicates (*n* = 17-18/group) (c). The survival curves of the vehicle control, 15% MS-20, anti-PD1 and 15% MS-20 plus anti-PD1 groups were determined (*n* = 12 mice/group) (d). CD62L^−^CD44^+^CD8^+^ T cells were determined using flow cytometry (*n* = 6/group) (e). IHC staining revealed PD1^+^ signals in the vehicle control, 15% MS-20, anti-PD1 and 15% MS-20 plus anti-PD1 groups. The number of cells in whole tumor tissue was quantified using HALO software, and the percentage of PD1^+^ cells was calculated as the sum of PD1^+^ cells divided by the total number of nuclear cells (*n* = 10/group) (f). Tumor weights of the vehicle control, 15% MS-20 plus anti-PD1 antibody and 15% MS-20 plus anti-PD1 and anti-CD8 antibody groups. (*n* = 11-12/group) (g). The data represent the mean±sd (b, c, e, f, g). The differences were assessed using Student’s t test (b, c, e, f, g) or the log-rank test (d). *p ≤ 0.05, **p ≤ 0.01 and ***p ≤ 0.001.

We first utilized a mouse CT-26 xenograft model in a prevention-based scenario to assess the combined effects of MS-20 and an anti-PD1 antibody on the treatment of colon cancer (Figure S1a). The mice were orally treated with vehicle control or with 1%, 5% or 15% MS-20 every day during the experiment beginning on day 0, when 2 × 10^5^ CT-26 cells were implanted. On days 6, 8, 10, and 12, the mice were *i.p*. administered an anti-PD1 antibody. Consistent with previous studies, anti-PD1 therapy alone caused a significant but modest reduction in the tumor volume compared to that in the vehicle control group (Figure S1b). Interestingly, the magnitude of tumor growth inhibition appeared to be proportional to the dose of MS-20 in the groups cotreated with the anti-PD1 antibody (Figure S1b). We further tested the effect of MS-20 in a scenario similar to that observed in the clinic. As depicted in Figure 1a, MS-20 was administered daily after grouping, and the anti-PD1 antibody was administered via *i.p*. injection at the indicated time points. Cotreatment with MS-20 and the anti-PD1 antibody significantly reduced the tumor growth by 40%-70% compared with that in the vehicle control and anti-PD1 antibody only treatment groups (Figure 1b). Similarly, the average tumor volume was also significantly lower in the cotreatment group than in the vehicle control and anti-PD1 monotherapy groups (Figure 1c). To clarify the antitumor effect of MS-20 alone, mice were randomly assigned to receive the vehicle control or 1%, 5% or 15% MS-20 orally every day after tumor growth (Figure S1c). Tumor growth did not differ significantly between groups treated with different doses of MS-20 and the vehicle control group (Figure S1d). The survival of the BALB/c mouse colon cancer model was also monitored for 28 days, and the mice receiving both MS-20 and the anti-PD1 antibody had the highest survival rate compared to the vehicle control and anti-PD1 alone groups (Figure 1d). In addition to its effect on colon cancer treatment, we investigated the effect of MS-20 on the efficacy of anti-PD1 antibody in a lung cancer animal model (Figure S1e). Similarly, cotreatment with MS-20 and an anti-PD1 antibody significantly reduced tumor growth and weight compared to those in the vehicle control and anti-PD1 antibody alone groups (Figure S1f-g).

Next, we examined tumor-infiltrating CD8^+^ T cells that play a critical role in the antitumor effects of immunotherapy. While treatment with the anti-PD1 antibody alone tended to increase CD8^+^ signals, cotreatment with MS-20 and the anti-PD1 antibody further increased CD8^+^ signals in the tumor microenvironment compared to those in the vehicle control group, as shown by immunohistochemistry (Figure S1h). An increasing trend for CD8^+^ signals after the administration of anti-PD1 alone or in combination with MS-20 was also observed using immunofluorescence staining (Figure S1i). We further investigated the function of tumor-infiltrating CD8^+^ T cells by analyzing CD62L^−^CD44^+^ T cells to identify effector memory CD8^+^ T cells, which were previously shown to exhibit upregulated expression of cytotoxic markers, including tumor necrosis factor-α (TNF-α), granzyme B and perforin, compared to central memory CD8^+^ T cells and to exhibit antitumor activity.^25^ Treatment with MS-20 combined with an anti-PD1 antibody significantly increased the effector memory CD8^+^ T cells subpopulation compared to that in the vehicle control and anti-PD1 group (Figure 1e). In addition, PD1, which acts as an immunosuppressive marker in immunotherapy, was also examined. PD1^+^ cells were not inhibited by anti-PD1 therapy alone, while cotreatment with MS-20 significantly inhibited PD1^+^ cells (Figure 1f). These data suggested that anti-PD1 antibody increased total CD8^+^ T cells while addition of MS-20 to anti-PD1 antibody further modulated the immunologic tumor microenvironment by increasing activated CD8^+^ T cells population and downregulating the expression of the immune exhaustion marker PD1. Last, an anti-CD8 neutralizing antibody was utilized to deplete the CD8^+^ T-cell population and to confirm the role of these cells in the antitumor activity of the MS-20 plus anti-PD1 treatment. Remarkably, the tumor inhibitory effect of the combined treatment was completely abrogated in the presence of the anti-CD8 neutralizing antibody (Figure 1g).

Collectively, these data indicated that oral administration of MS-20 could enhance the antitumor activity of the anti-PD1 antibody and prolong mouse survival. The addition of MS-20 to anti-PD1 antibody further increased effector memory CD8^+^ T cells subpopulation and downregulated immune exhaustion marker PD1.

### The MS-20-induced enrichment of the gut microbiota enhanced the efficacy of the anti-PD1 antibody treatment

We collected fecal samples from the vehicle, MS-20, anti-PD1 antibody, and MS-20 plus anti-PD1 antibody groups and subsequently analyzed the microbiota composition to assess the impact of MS-20 on the gut microbiota. Although the α-diversity did not differ among the four groups (Figure S2a), the β-diversity of the MS-20 plus anti-PD1 antibody group differed significantly from that of the other groups (Figure S2b). The taxonomy of the bacteria was distinct between groups, especially in the MS-20 plus anti-PD1 antibody group (Figure S2c). Compared to the vehicle control, the MS-20 treatment modestly increased the abundance of Ruminococcaceae and Bacteroidaceae. Compared with those in the anti-PD1 antibody group, the abundances of Lachnospiraceae and Oscillospiraceae decreased, while the abundances of Lactobacillaceae and Tannerellaceae increased in the MS-20 plus anti-PD1 antibody group (Figure S2c). Linear discriminant analysis effect size (LEfSe) was performed to identify bacteria with different abundances between different groups. The fecal microbial profiles were compared between the vehicle control group and the MS-20 group or between the MS-20 plus anti-PD1 group and anti-PD1 antibody alone group (Figure S2d-e). *Lactobacillus murinus*, a member of Lactobacillaceae, and Tannerellaceae were more abundant in the MS-20 plus anti-PD1 antibody group than in the anti-PD1 antibody group. Similarly, the abundances of *Clostridium colinum* and *Tyzzerella* spp., which belong to Lachnospiraceae, decreased, and the abundance of *Clostridium leptum*, which belongs to Oscillospiraceae, decreased in the MS-20 and MS-20 plus anti-PD1 antibody groups compared to the control and anti-PD1 antibody groups, respectively (Figure S2d-e).

**Figure 2.**
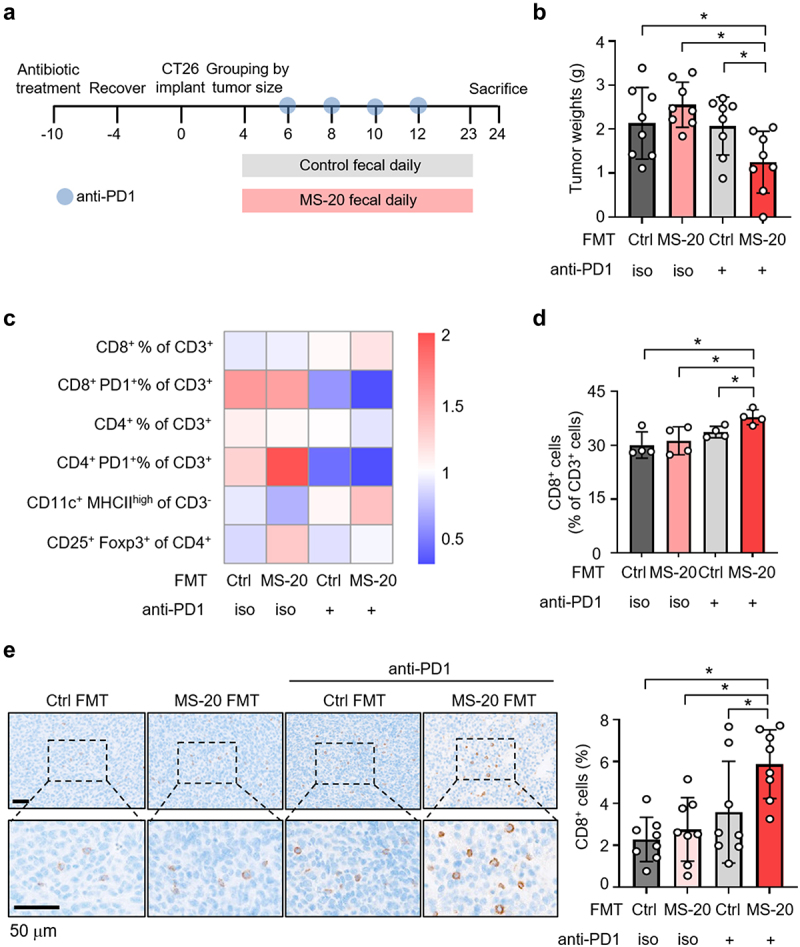
MS-20-treated feces enhanced the efficacy of the anti-PD1 antibody.

**Note**: Drinking water containing antibiotics (ampicillin, neomycin, metronidazole and vancomycin) was orally administered to the mice to remove the gut bacteria. After tumor growth, the mice were randomly assigned to receive FMT of vehicle control or MS-20. The anti-PD1 antibody was administered at the indicated time points (a). The tumor weights of the control FMT and MS-20 FMT groups treated with or without the anti-PD1 antibody were determined (*n* = 8/group) (b). The profile of tumor-infiltrating lymphocytes was determined using flow cytometry. The data are presented as the mean percentages of four mice and normalized by mean. (c). CD8^+^ T cells from the groups receiving the control FMT and MS-20 FMT with or without cotreatment with an anti-PD1 antibody were analyzed via flow cytometry (*n* = 4/group) (d). IHC staining was used to assess CD8^+^ T-cell signals from control FMT and MS-20 FMT mice treated with or without an anti-PD1 antibody. The number of cells in the whole tumor tissue was quantified using HALO software, and the percentage of CD8^+^ cells was calculated as the sum of CD8^+^ cells divided by the total number of nuclear cells (*n* = 8/group) (e). The data are presented as the mean±sd (b, d, e) or mean (c). The differences were assessed using Student’s t test (b, d, e). *p ≤ 0.05, **p ≤ 0.01 and ***p ≤ 0.001.

We next conducted an FMT experiment with a mouse model of colon cancer to further investigate the effect of the MS-20-treated gut microbiota on tumor immunotherapy efficacy. Initially, mice were orally administered drinking water containing a consortium of antibiotics to deplete the gut microbiota. Later, the mice were subcutaneously inoculated with CT-26 tumor cells and randomly assigned to four groups after tumor growth.^3^ Control- or MS-20-treated fecal microbiota were orally administered to mice daily, and the anti-PD1 antibody was injected at the indicated time points (Figure 2a). A significant reduction in tumor weight was observed in the mice receiving feces derived from the MS-20-treated donors in combination with the anti-PD1 antibody compared to the other groups (Figure 2b). In addition, we analyzed the infiltration of various types of immune cells in the tumor microenvironment of recipient mice using flow cytometry. Compared to the other treatments, MS-20 FMT combined with an anti-PD1 antibody significantly increased the infiltration of total CD8^+^ T cells (Figure 2c-d) and tended to increase the proportion of CD11c^+^ MHCII^high^ dendritic cells (Figure 2c and Figure S3a). In comparison, no differences in the percentages of CD4^+^CD25^+^Foxp3^+^ Treg cells or total CD4^+^ T cells were observed among the groups (Figure 2c and Figure S3b). As PD1 has an immunosuppressive role, we next examined the percentage of PD1-positive cells among the T-cell subpopulations. CD8^+^PD1^+^ T cells appeared to decrease after anti-PD1 treatment but only reached statistical significance in the mice that also received MS-20-treated feces (Figure 2c and Figure S3c), whereas CD4^+^PD1^+^ T cells were also decreased by anti-PD1 treatment and displayed a greater decrease in the MS-20 FMT plus anti-PD1 antibody group (Figure S3d). Moreover, the increase of the percentage of CD8^+^ T cells in the combination group was further confirmed by immunohistochemistry (Figure 2e). Taken together, the inhibition of tumor growth and increase in the tumor-infiltrating CD8^+^ T cells could be attributed to the enhanced anti-PD1 activity of the MS-20-treated gut microbiota.

### Analysis of the microbiota composition after MS-20 FMT

**Figure 3.**
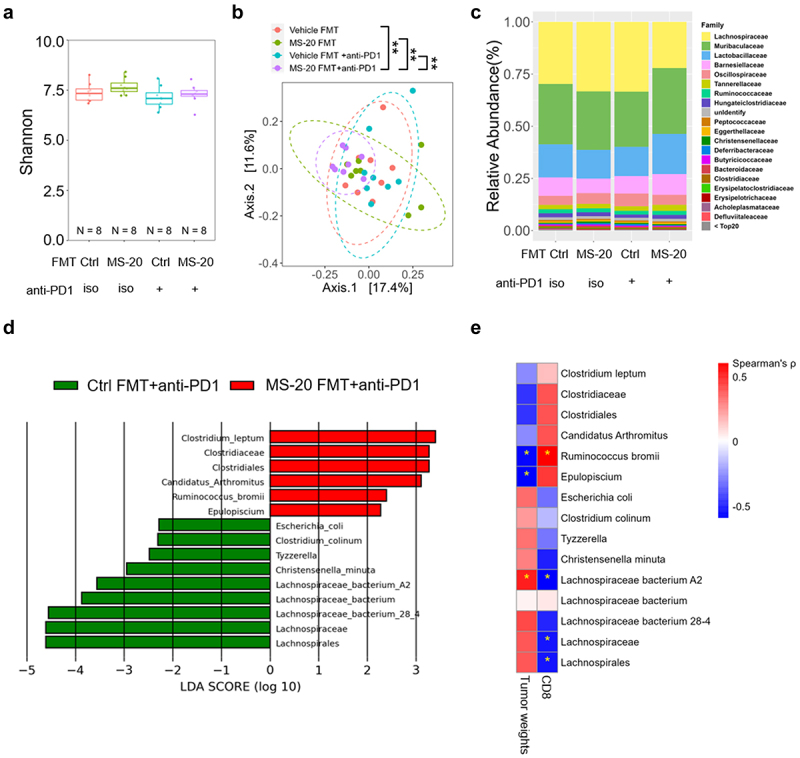
MS-20 distinctly modulated the gut microbiota associated with tumor reduction and CD8^+^ T-cell infiltration.

**Note**: A 16S RNA sequence analysis was performed to assess the α-diversity (a) and β-diversity (b) of control FMT and MS-20 FMT samples with or without cotreatment with the anti-PD1 antibody. The bacterial compositions of the control FMT and MS-20 FMT samples with or without cotreatment with the anti-PD1 antibody are shown in (c). The key bacteria contributing to anti-PD1 efficacy were determined using LEfSe and compared between the control FMT and MS-20 FMT combined with anti-PD1 cotreatment groups. Bacteria with LDA scores ≥ 2 are shown (d). The correlations of MS-20-regulated bacteria with tumor reduction and CD8^+^ T-cell infiltration were evaluated. A star represents a significant difference (e). The differences were assessed using Student’s t test (a) and permutation testing (b). Spearman’s rank correlation coefficient (ρ) was calculated to assess the relationships among groups (e). *n* = 8/group. *p ≤ 0.05, **p ≤ 0.01 and ***p ≤ 0.001.

Next, we assessed the gut microbiota composition of the recipient mice after FMT. The α-diversity did not differ among the four groups, while the β-diversity of the MS-20-induced fecal microbiota plus anti-PD1 antibody group differed significantly from that of the other groups (Figure 3a-b). A taxonomic analysis of the bacteria also revealed a distinct bacterial composition in the MS-20 FMT plus anti-PD1 antibody group (Figure 3c). Specifically, the abundance of Lachnospiraceae decreased, while the abundances of Lactobacillaceae, Barnesiellaceae, and Tannerellaceae increased in the MS-20 FMT plus anti-PD1 antibody group (Figure 3c). LEfSe revealed that Clostridiales, *Clostridium leptum*, *Candidatus arthromitus* and *Ruminococcus bromii* were increased in the MS-20 FMT plus anti-PD1 antibody group compared to the anti-PD1 antibody alone group (Figure 3d). In comparison, the abundances of *Clostridium colinum* and *Tyzzerella* spp., which belong to Lachnospiraceae, decreased (Figure 3d). This finding was in accordance with the results of a previous study in which Clostridiales was positively associated with tumor-infiltrating immune cells, such as CD3^+^ and CD8^+^ cells, in melanoma patients.^6^ Additionally, *R. bromii* is enriched in hepatocellular carcinoma (HCC) and non-small cell lung cancer (NSCLC)^Akk+^ patients who respond to immunotherapy. ^26–29^

Bacteria identified by LEfSe (Figure 3d) were analyzed by pairwise comparisons using nonparametric Spearman’s correlations with either tumor weight (Figure 2b) or CD8^+^ cell abundance (Figure 2d) to reveal the correlation between the gut microbiota and antitumor activity. Interestingly, MS-20 plus anti-PD1 antibody-enriched bacteria were negatively correlated with the tumor weight but positively correlated with CD8^+^ T cells in the tumor microenvironment. On the other hand, bacteria with decreased abundance following treatment with MS-20 plus an anti-PD1 antibody were positively correlated with the tumor weight but negatively correlated with tumor-infiltrating CD8^+^ T cells (Figure 3e). Among these bacteria, *Epulopiscium* spp. and *R. bromii*, which were enriched in the MS-20 plus anti-PD1 antibody group, exhibited significant negative correlations with the tumor weight. Furthermore, *R. bromii* was positively correlated with intratumoral CD8^+^ T cells (*p* < 0.05). Notably, we also found that the abundance of *R. bromii* tended to increase in mice that were orally administered MS-20 independent of anti-PD1 treatment (Figure S4a). In addition, *R. bromii* was also enriched in mice receiving FMT from MS-20-treated donors in the presence or absence of anti-PD1 cotreatment (Figure S4b). In contrast, Lachnospiraceae bacterium A2 was positively correlated with tumor weight, while Lachnospiraceae and Lachnospiraceae bacterium A2 were negatively correlated with CD8^+^ T cells in the tumor microenvironment (Figure 3e). Taken together, these data indicated that the distinct microbiota compositions shaped by MS-20 play a critical role in enhancing the effectiveness of anti-PD1 antibody treatment. Key bacterial species, such as *R. bromii*, were correlated with a reduced tumor weight and the infiltration of CD8^+^ T cells in the tumor microenvironment.

### MS-20 regulated intestinal homeostasis and immunity

**Figure 4.**
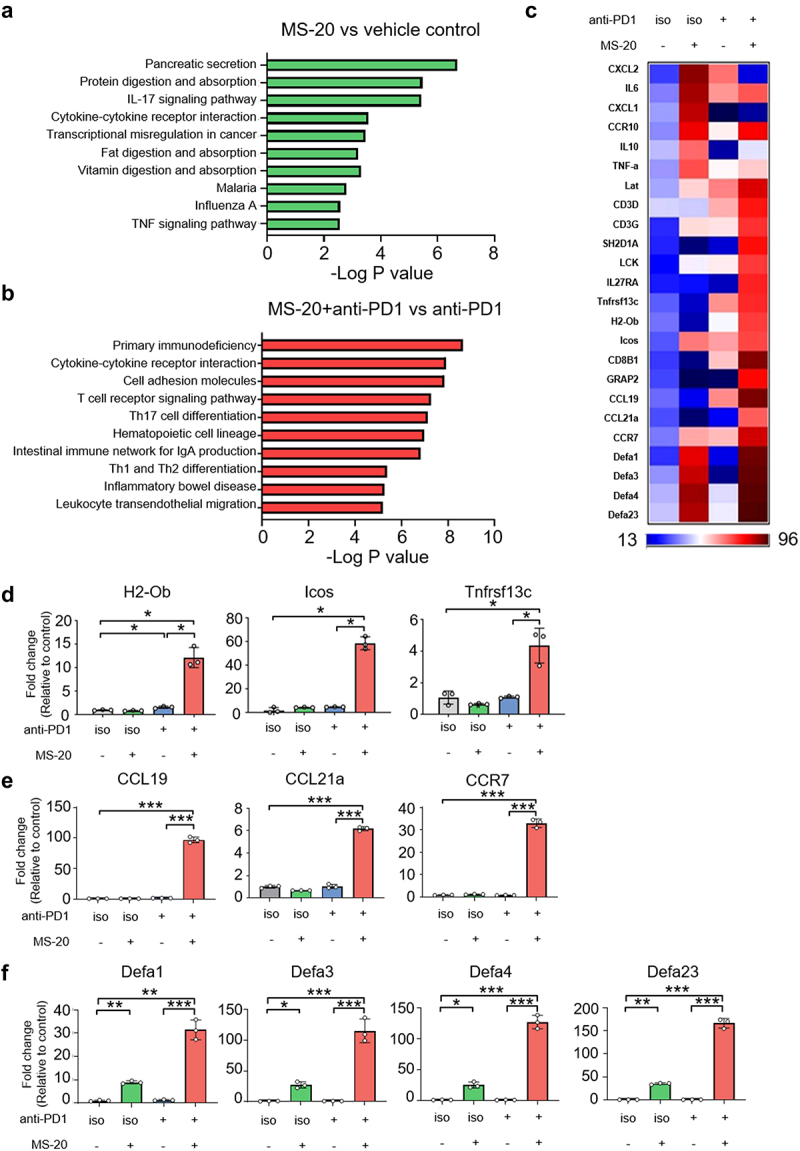
Intestinal immunity was enriched after MS-20 treatment.

**Note**: KEGG-predicted enriched pathways associated with MS-20 alone and the vehicle control (a), and MS-20 combined with anti-PD1 and anti-PD1 antibody alone (b) are shown. The heatmap shows the DEGs between the vehicle control, 15% MS-20, anti-PD1 and 15% MS-20 groups combined with the anti-PD1 antibody (c). The expression of genes related to IgA signaling (*n* = 3/group) (d), chemoattractant cytokines produced by immune cells (*n* = 3/group) (e) and defensins (*n* = 3/group) (f) was determined using RT-qPCR. The data are presented as the mean±sd (d, e, f). The differences were assessed using Student’s t test (d, e, f). *p ≤ 0.05, **p ≤ 0.01 and ***p ≤ 0.001.

As MS-20 was orally administered to mice, we investigated the effects of MS-20 on the messenger RNA expression pattern in mouse colon tissue via a transcriptomic analysis. Compared to those in the vehicle control group, the IL-17 signaling pathway, cytokine-cytokine receptor interaction pathway and TNF signaling pathway were significantly upregulated in the MS-20-treated group, based on the KEGG network analysis (Figure 4a). Compared to those in the anti-PD1 group, the genes involved in cell adhesion molecules, the T-cell receptor signaling pathway, the intestinal immune network for IgA production and the leukocyte transendothelial migration pathway were increased in the MS-20 plus anti-PD1 antibody treatment group (Figure 4b). These data suggested that the MS-20 plus anti-PD-1 antibody treatment might regulate the maturation and homing of immune cells in the intestine. The hierarchical clustering of differentially expressed genes (DEGs) in different experimental groups is shown in Figure 4c. Genes upstream of the IgA production signaling pathway, such as H2-Ob, which encodes subunits of MHC in dendritic cells (DCs); Icos, which is important for CD4 activation; and Tnfrs13c, which encodes the B-cell-activating factor receptor, were expressed at higher levels in the MS-20 plus anti-PD1 group than in the vehicle control and anti-PD1 groups (Figure 4d). Moreover, the expression of Grap2 and Lat, which encode proteins involved in the activation of T cells, was upregulated in the MS-20 plus anti-PD1 group compared to the vehicle control group (Figure S5).

The expression of two CCR7 ligands (CCL19 and CCL21a) and CCR7 was upregulated in the combination therapy group (Figure 4e). CCR7 is normally expressed on double-negative and single-positive thymocytes (naïve T cells, central memory T cells, regulatory T cells, naïve B cells, semimature/mature DCs and NK cells). CCR7 acts as a critical regulator that guides homeostatic lymphocytes to secondary lymphoid organs.^30,31^ A previous study revealed that CCL19 and CCL21 are potent inducers of DC maturation and migration.^32,33^ Additionally, the CCL19/CCL21-CCR7 axis is critical for T and B-cell homing.^34^ These data suggested that MS-20 might regulate cytokines for immune cell attraction in colon cells, but additional experiments are needed to confirm these findings. Moreover, the gene expression levels of Defa1, Defa3, Defa4 and Defa23, which belong to the α-defensin family, were increased by MS-20 alone or the MS-20 plus anti-PD1 treatment (Figure 4f). Notably, human α-defensins are also chemoattractants for multiple immune cell types, such as immature dendritic cells, macrophages, and mast cells.^35^ Overall, we found that the oral administration of MS-20 alone or in combination with an anti-PD1 antibody affected the intestinal immune environment.

### MS-20 modulated immunotherapy-related and colon cancer-related pathological bacteria ex vivo

An *ex vivo* fecal culture system established for studying the microbiota profile *in vitro*^36^ was used to investigate whether MS-20 could regulate the microbiota composition of cancer patients. Stool samples were collected from seven colorectal cancer patients, followed by treatment with the vehicle control or MS-20 for 24 h. α-diversity was not significantly different between the vehicle and MS-20 groups (Figure S6a). The β-diversity analysis revealed significant dissimilarity, with the MS-20 group exhibiting distinct microbial communities compared to those of the vehicle control group (Figure 5a). MS-20 treatment resulted in a distinct microbial composition from that in the vehicle samples at the family level (Figure 5b). In the presence of MS-20, the abundances of Lachnospiraceae, Ruminococcaceae, Bifidobacteriaceae and Veillonellaceae increased, while those of Bacteroidaceae, Eggerthellaceae and Peptostreptococcaceae decreased. The differential abundance analysis revealed that the abundances of several immunotherapy responder-associated bacteria, including *Faecalibacterium prausnitzii, Blautia wexlerae, Fusicatenibacter saccharivorans* and *Bifidobacterium longum*, increased after MS-20 treatment. In contrast, the abundance of nonresponder-associated bacteria, including *Hungatella hathewayi* and *Clostridium symbiosum*, decreased after MS-20 treatment (Figure 5c).^37,38^ In addition, the abundances of *Bilophila wadsworthia* and *Enterocloster aldensis*, which are enriched in colon cancer patients, decreased in the MS-20 group (Figure 5c).^39,40^ We also collected eight stool samples from NSCLC patients for analysis. Similarly, the abundances of *F. prausnitzii*, *F. saccharivorans* and *Agathobaculum butyriciproducens* increased after MS-20 treatment (Figure S6b). These results collectively suggested that MS-20 regulates bacteria associated with the immunotherapy response and pathobionts that may be involved in colon cancer pathology. RT-qPCR was utilized to determine whether a correlation existed between the bacteria identified and MS-20 treatment. MS-20 administration significantly increased the abundances of *F. prausnitzii*, *F. saccharivorans* and *B. longum* (Figure 5d), and the abundances of *F. prausnitzii* and *B. longum* exhibited dose-response effects (linear mixed regression model, *p* = 0.021 and *p* = 0.005, respectively). We further analyzed microbial co-occurrence networks to investigate the interactions between the vehicle control and MS-20 treatment groups (Figure S6c). After MS-20 treatment, *R. bromii* was positively correlated with *Parasutterella excrementihominis*, *Roseburia inulinivorans* and *F. saccharivorans*, but these correlations were not found in the vehicle control group (Figure 5e). Among the identified bacteria, *R. inulinivorans* and *F. saccharivorans* were associated with the immunotherapy response.^37^ In the aforementioned animal studies, the abundance of *R. bromii* tended to increase in mice that received MS-20 orally or via FMT from MS-20-treated donors (Figure 3d and Figure S4). Here, we also observed elevated levels of *R. bromii* in the feces of cancer patients after MS-20 treatment *ex vivo* (Figure 5f), which suggested that *R. bromii* might be one of the critical bacteria affected by MS-20.

**Figure 5A.**
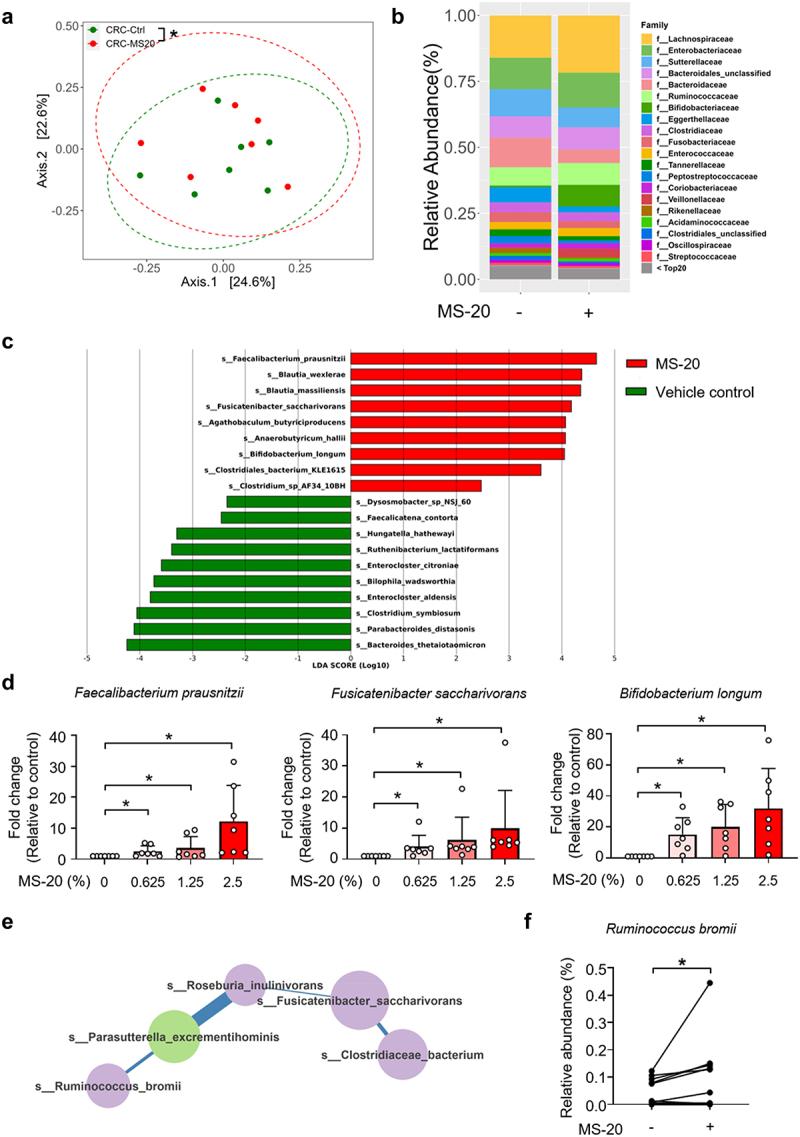
MS-20 regulated the microbiota of cancer patients *ex vivo*.

The feces of colon cancer patients were treated with the vehicle control or 2.5% MS-20 for 24 h, and the microbiota was analyzed via metagenomics analysis. β-diversity of the control and 2.5% MS-20 groups was assessed (a). The bacterial composition at the family level was determined in the control and 2.5% MS-20 groups (b). LEfSe was used to identify differentially abundant bacteria between the vehicle control and 2.5% MS-20 groups. Bacteria with LDA scores ≥ 2 are shown (c). The feces of colon cancer patients were treated with the indicated dose of MS-20 for 24 h, and immunotherapy response-associated bacteria were examined using RT-qPCR (*n* = 7/group) (d). The co-occurrence networks of the vehicle control and MS-20 treatment groups were analyzed, with a specific focus on the 0.5% prevalence threshold. The data revealed an *R. bromii-*dominant network following MS-20 treatment (e). The relative abundance of *R. bromii* in cancer patients after MS-20 treatment was determined (*n* = 15/group) (f). The MetaCyc analysis predicted metabolic pathways in fecal samples from individuals with CRC treated with or without MS-20. LEfSe was used to determine linear discriminant analysis (LDA) scores for differentially abundant pathways in the vehicle control versus MS-20 treatment groups. A heatmap was generated to show the abundance of each outcome-associated pathway in each sample; the largest value was defined as the maximum, and the smallest value was defined as the minimum (*n* = 7/group) (g). The relative abundance of significant metabolic pathways were determined. The relative abundance of metabolic pathways that were classified in the same category according to MetaCyc were added together and are shown for individual samples. Each dot represented an individual sample. (*n* = 7/group) (h-i). The data are presented as the mean±sd (d, f, h, i). The differences were assessed using permutation testing (a), the Wilcoxon rank sum test (d), the Wilcoxon signed rank test (f) or Student’s t test (h, i). *p ≤ 0.05, **p ≤ 0.01 and ***p ≤ 0.001.

A MetaCyc analysis was subsequently performed, which revealed several pathways that were altered by MS-20 treatment (Figure 5g). Among these pathways, we detected increases in the thiamine diphosphate biosynthesis and palmitate biosynthesis pathways following MS-20 treatment (Figure 5h). A previous study identified a microbiota hazard score associated with the stage and survival of patients with colon cancer, where the thiamine diphosphate biosynthesis and palmitate biosynthesis pathways were enriched in patients with a low risk of colon cancer.^41^ Conversely, the histidine degradation and B6 biosynthesis pathways were downregulated by MS-20 treatment (Figure 5i), and the histidine degradation pathway was decreased in patients with a low colon cancer risk.^41^ Briefly, these data suggested that MS-20 regulates metabolic pathways, skewing toward a profile associated with a decreased risk of colon cancer. For NSCLC, the MetaCyc analysis revealed that MS-20 regulated immunotherapy-associated metabolic pathways (Figure S6d). The pathway involved in guanine nucleotide degradation and purine degradation decreased after MS-20 treatment. On the other hand, L-isoleucine biosynthesis and branched amino acid biosynthesis were upregulated in the MS-20 group (Figure S6e). Interestingly, in a fecal analysis of NSCLC patients who received immunotherapy alone or in combination with chemotherapy and experienced longer overall survival, the nucleoside and nucleotide degradation pathways were inhibited, and the metabolic pathway involved in the amino acid biosynthesis pathway was upregulated.^42^ In summary, MS-20 modulated the microbial composition and immunotherapy response-associated bacteria in cancer patients *ex vivo*. The predicted metabolic pathways of MS-20-regulated microbiota might be associated with a lower risk of advanced stages of CRC and a better response to immunotherapy in NSCLC patients.

**Figure 5B.**
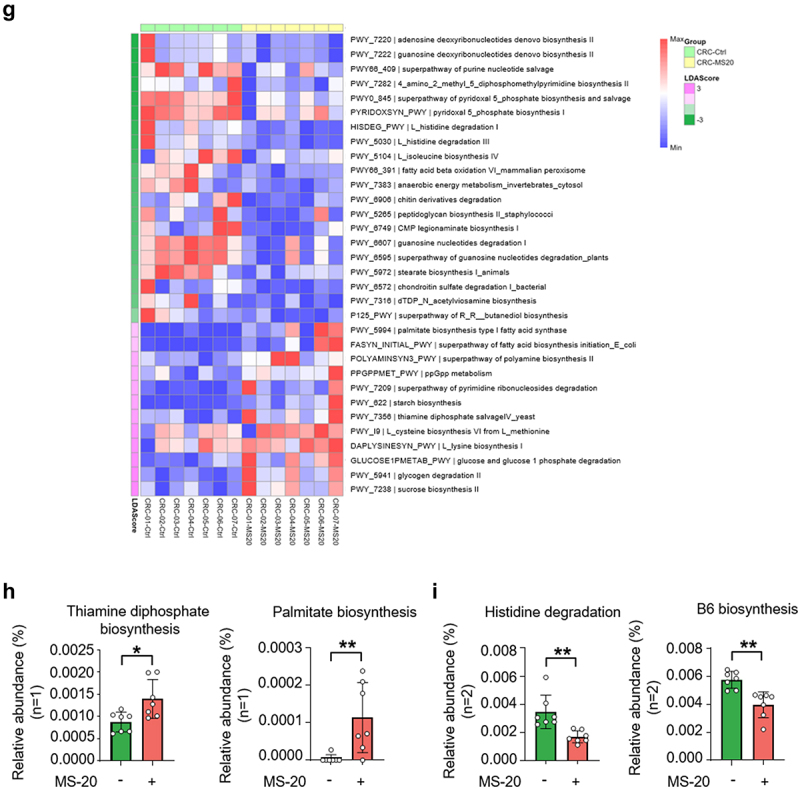
continued.

## Discussion

In this study, MS-20 treatment in combination with an anti-PD1 antibody effectively inhibited tumor growth, increased the abundance of total and activated CD8^+^ T cells and inhibited PD1 expression in the tumor microenvironment in animal models. Moreover, FMT experiments in mice confirmed that MS-20-mediated regulation of the microbiota enhanced the efficacy of the anti-PD1 antibody treatment and promoted intratumoral CD8^+^ T-cell infiltration. MS-20 might regulate intestinal immunity by modulating the gut microbiota composition in animals. In addition, we also found that MS-20 regulated the microbial composition in the feces of cancer patients *ex vivo*, where the enrichment of bacteria and their predicted metabolic activities may resemble the gut microbiota profile of individuals who respond to immunotherapy.^37^ Briefly, MS-20, a fermented soybean extract, enhanced the efficacy of the anti-PD1 antibody through the regulation of complex interactions between the microbiota and immune cells and intestinal cells. These findings suggested that MS-20 can act as a potential adjuvant in combination with an anti-PD1 antibody to enhance the efficacy of immunotherapy.

While efforts are being made to increase the efficacy of immunotherapy by modulating the gut microbiota through probiotics, prebiotics or FMT, challenges still remain. For example, intensive studies on whether live biotherapeutic products (LBPs) could work for different populations worldwide and how to specifically identify the optimal sites for LBPs to elicit their effects have also been conducted.^43^ Furthermore, live bacteria pose infection risks, particularly in immunocompromised individuals.^44,45^ Additionally, FMT from responders or healthy donors to nonresponder patients prolongs the progression-free survival of cancer patients.^16^ However, the source, preparation of feces, scale, and safety of these treatments still need to be assessed.^11^ Therefore, additional strategies that can modulate the gut microbiota are still needed. MS-20 is a soybean-fermented broth produced through anaerobic fermentation using a distinctive combination of bacteria. MS-20 undergoes heat inactivation and filtration processes to preserve microbial metabolites and is a postbiotic that has emerged as a promising strategy for improving human health.^46^ The distinct advantage of this fermented product is that, unlike live bacterial products that rely on colonization for effectiveness, its impact seems to not be affected by variations in the individual’s intestinal environment. Additionally, the stability of probiotics is a concern, as they are often sensitive to stomach acid and temperature variations (storage conditions).^47^ Unlike traditional probiotics, MS-20, which is safe and unaffected by changes in temperature, stomach acidity, and digestive enzymes, has robust stability.^48^

We found that MS-20 in combination with an anti-PD1 antibody inhibited tumor growth and increased the total and functional CD8^+^ T cells. Our fecal transplantation animal study also revealed that transplanting feces from MS-20-treated mice in combination with an anti-PD1 antibody enhanced antitumor activity, promoted tumor infiltration of CD8^+^ T cells and decreased suppressive CD8^+^PD1^+^ T cells subpopulation (Figure 2b-d and Figure S3c). These findings suggested that the immune modulation achieved by the MS-20-regulated microbiota may be an important factor leading to enhanced function of the anti-PD1 antibody. The differential analysis revealed that MS-20 FMT plus the anti-PD1 antibody increased Clostridiales and *R. bromii* abundances while decreasing the Lachnospiraceae abundance (Figure 3d). These results are consistent with previous studies showing that microbial organisms influence the response to immune checkpoint inhibitors and immune cells in the tumor microenvironment.^29^ For instance, Clostridiales is positively correlated with increased intratumour CD8^+^ T cells and granzyme B expression in individuals with melanoma.^6^ On the other hand, an increased abundance of Ruminococcaceae is observed in patients who respond to immunotherapy for NSCLC and HCC.^49,50^ The *ex vivo* platform was also utilized to mimic the microbial changes induced by MS-20 in humans, and MS-20-modulated bacteria and metabolic pathways were associated with the response to immunotherapy (Figure 5). However, we observed that the microbial profile differed between mice and humans, possibly because only a handful of species are shared between mice and humans,^51^ and the gut microbiota of mice has not been fully characterized.^52^ Nevertheless, *R. bromii*, is one of the gut bacteria that is responsive to MS-20 administration *in vivo* (Figure S4), also participated in the network of bacteria associated with the immunotherapy response in the feces of cancer patients according to the *ex vivo* results (Figure 5e-f). *R. bromii* is known for its critical function in degrading resistant starch in the human colon and was found at an evaluated level in patients with metastatic melanoma who responded to anti-PD1 inhibitors.^6,53^ Moreover, a recent study by Messaoudene et al. showed that castalagin, a bioactive polyphenol from the berry camu-camu (*Myrciaria dubia*), was enriched in bacteria (including Ruminococcaceae) associated with efficient immunotherapeutic responses and improved the CD8^+^/Foxp3^+^CD4^+^ T-cell ratio within the tumor microenvironment.^54^ Oral supplementation of mice with castalagin following FMT from immunotherapy-refractory patients promoted an antitumor response to the anti-PD-1 antibody.^54^ In a proof-of-principle experiment analyzed the feces of two noncancer patients enrolled in a clinical trial (NCT04058392) to determine the safety of oral administration of *M. dubia*. Preliminary fecal metagenomics revealed a positive trend for diversity and toward the enrichment of *R. bromii*, consistent with the results obtained from *M. dubia-*treated mice.^54^ Briefly, these findings support the idea that the enhanced antitumor function of the anti-PD1 antibody may be mediated through the modulation of the gut microbiota by MS-20.

According to the transcriptomic analysis of colon epithelial cells, MS-20 plus an anti-PD-1 antibody increased the expression of genes such as H2-Ob, Icos and Tnfrs13c, which are related to IgA production (Figure 4d). IgA can target multiple microbial antigens, including LPS, proteins, flagellin and virulence factors (exotoxins), from pathogenic microbes; therefore, IgA is critical for maintaining a symbiotic balance between the microbiota and the immune system.^55–58^ In addition, gut microbiota alterations were also observed in humans with IgA deficiency.^59–61^ Conversely, gut microbiota commensals have also been reported to regulate IgA levels. For example, oral administration of the probiotic bacterium *Lactobacillus casei* to mice increased the number of IgA^+^ cells in the small intestinal lamina propria.^62^ Accordingly, we also observed an increase in Lactobacillaceae in the MS-20 plus anti-PD1 antibody group (Figure S2e), consistent with the finding that these bacteria are involved in the increase in IgA levels.^61^ In addition, a previous study revealed that IgA deficiency promoted the development of colorectal cancer induced by AOM/DSS.^63^ Thus, the combined use of MS-20 and anti-PD-1 therapy very likely improves the function of immunoglobulins such as IgA and enhances the anticancer effect of these agents. Overall, the fine-tuning of the intestinal microbiota composition by MS-20 might be used to optimize immune checkpoint therapy and overcome the limitations of the original therapy, thus allowing MS-20 to become an adjuvant for cancer treatment.

**Figure 6.**
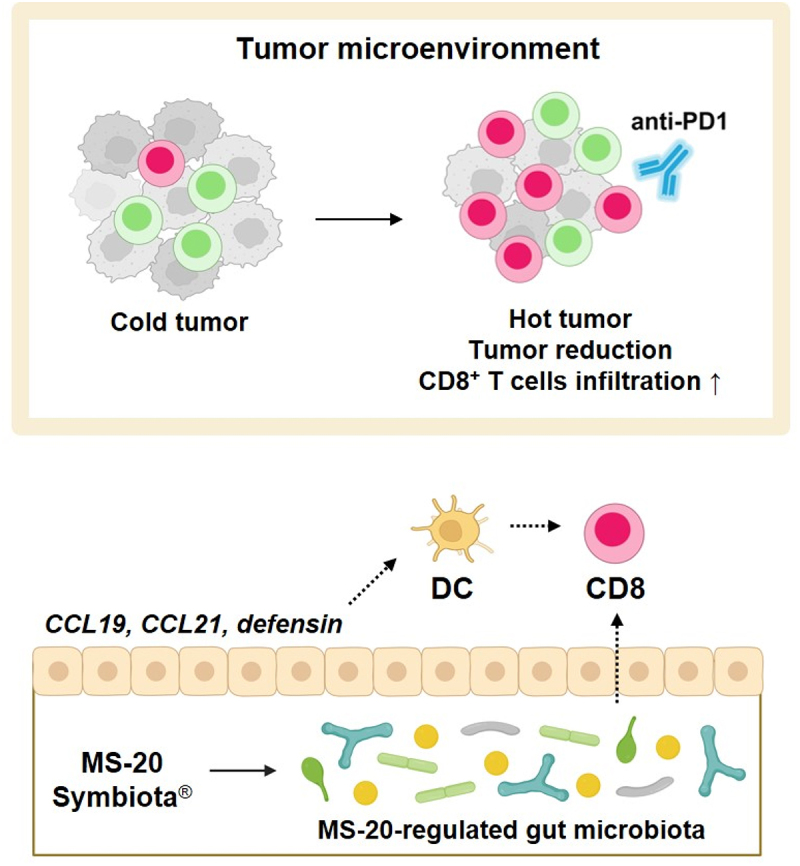
Proposed mechanism of the effect of MS-20.

**Note**: The symbiota MS-20 exhibited antitumor activity in combination with an anti-PD1 antibody. Treatment with MS-20 FMT enhanced the efficacy of anti-PD1 therapy and promoted the entry of CD8^+^ T cells into the tumor microenvironment. The abundances of distinct MS-20-regulated bacteria, such as *R. bromii*, were correlated with antitumor activity and CD8^+^ T-cell infiltration. MS-20 also regulated intestinal immunity through the upregulation of CCL19, CCL21 and defensin expression, which have been found to recruit and activate dendritic cells. These data suggested that MS-20 elicited antitumor activity in combination with anti-PD1 therapy by modulating the immune-associated gut microbiota and intestinal immunity. The figure was created with BioRender.com.

In summary, MS-20, in combination with an anti-PD1 antibody, inhibited tumor growth in animals. The distinct gut microbiota observed after treatment with MS-20 enhanced the efficacy of an anti-PD1 antibody and increased intratumoral CD8^+^ T cells, as shown in an FMT animal model. Furthermore, MS-20-regulated bacteria, such as *R. bromii*, were associated with tumor inhibition and CD8^+^ T-cell infiltration. MS-20 treatment also regulated intestinal immunity by enhancing the expression of genes such as CCL19, CCL21 and defensins, which are associated with DC recruitment and activation. In addition, using fecal samples from cancer patients, MS-20 modulated the abundance of immune checkpoint inhibitor (ICI)-responsive bacteria *ex vivo*. These findings suggested that MS-20 could be a promising adjuvant to turn cold into hot tumors and enhance the efficacy of immunotherapy (Figure 6).

## Supplementary Material

Revised Supplemental online material.docx

Supplemental online material.docx

## Data Availability

The data that support the findings of this study are available from the corresponding author upon reasonable request.
