# Supplementary material for: MS-20 enhances the gut microbiota-associated antitumor effects of anti-PD1 antibody
Source: Gut Microbes. 2024 Jul 30;16(1):2380061. doi: 10.1080/19490976.2024.2380061 (PMC11290773; doi:10.1080/19490976.2024.2380061)
Supplement: Revised Supplemental online material.docx [file KGMI_A_2380061_SM7480.docx]

**Supplemental online material**

**MS-20 enhances the gut microbiota-associated antitumor effects of anti-PD1 antibody**

**Authors**

Pei-Jung Lee^1^, Chien-Min Hung^1^, Ai-Jen Yang^1^, Cheng-Yu Hou^1^, Hung-Wen Chou^2^, Yi‐Chung Chang^3^, Wen-Cheng Chu^1^, Wen-Yen Huang^1^, Wen-Chih Kuo^1^, Chia-Chun Yang^3^, Li-Chun Chang^4^, Kang-Yun Lee^5^, Han-Pin Kuo^6,7^, Kung-Ming Lu^1^, Hsin-Chih Lai^8^, Ming-Liang Kuo^1^, and Wan-Jiun Chen^1*^

**Affiliations**

^1^Microbio Co., Ltd., Taipei, Taiwan

^2^Oneness Biotech Co., Ltd., Taipei, Taiwan

^3^Microbio (Shanghai) Biotech Company, Shanghai, China

^4^Division of Gastroenterology, Department of Internal Medicine, National Taiwan University Hospital, Taipei, Taiwan

^5^Division of Pulmonary Medicine, Department of Internal Medicine, Shuang Ho Hospital, Taipei Medical University, New Taipei City, Taiwan

^6^Pulmonary Medicine Research Center, Taipei Medical University, Taipei, Taiwan

^7^Department of Thoracic Medicine, Taipei Medical University Hospital, Taipei, Taiwan

^8^Revivebio Co., Ltd., Taipei, Taiwan

***Corresponding author**

Wan-Jiun Chen, PhD

Vice President, Microbio Co., Ltd.

Email: WanJiun.Chen@microbio.com.tw

Contact number: +886-2-2655-8558

Address: 14 F-1, No. 3 Yuan Qu St., Taipei, Taiwan

**Table S1 Patient characteristic**

| **Patient number** | **Gender** | **Age** | **Stage** |
| --- | --- | --- | --- |
| CRC-01 | M | 63.3 | II |
| CRC-02 | M | 67.4 | III |
| CRC-03 | M | 56.6 | III |
| CRC-04 | F | 72.7 | III |
| CRC-05 | M | 72.2 | III |
| CRC-06 | F | 35 | III |
| CRC-07 | F | 66.5 | III |
| NSCLC-01 | F | 64 | NA |
| NSCLC-02 | M | 64 | IVB |
| NSCLC-03 | M | 71 | NA |
| NSCLC-04 | M | 58 | NA |
| NSCLC-05 | M | 70 | IV |
| NSCLC-06 | M | 51 | IIIa |
| NSCLC-07 | M | 75 | IVB |
| NSCLC-08 | M | 64 | IVA |

NA: not applicable


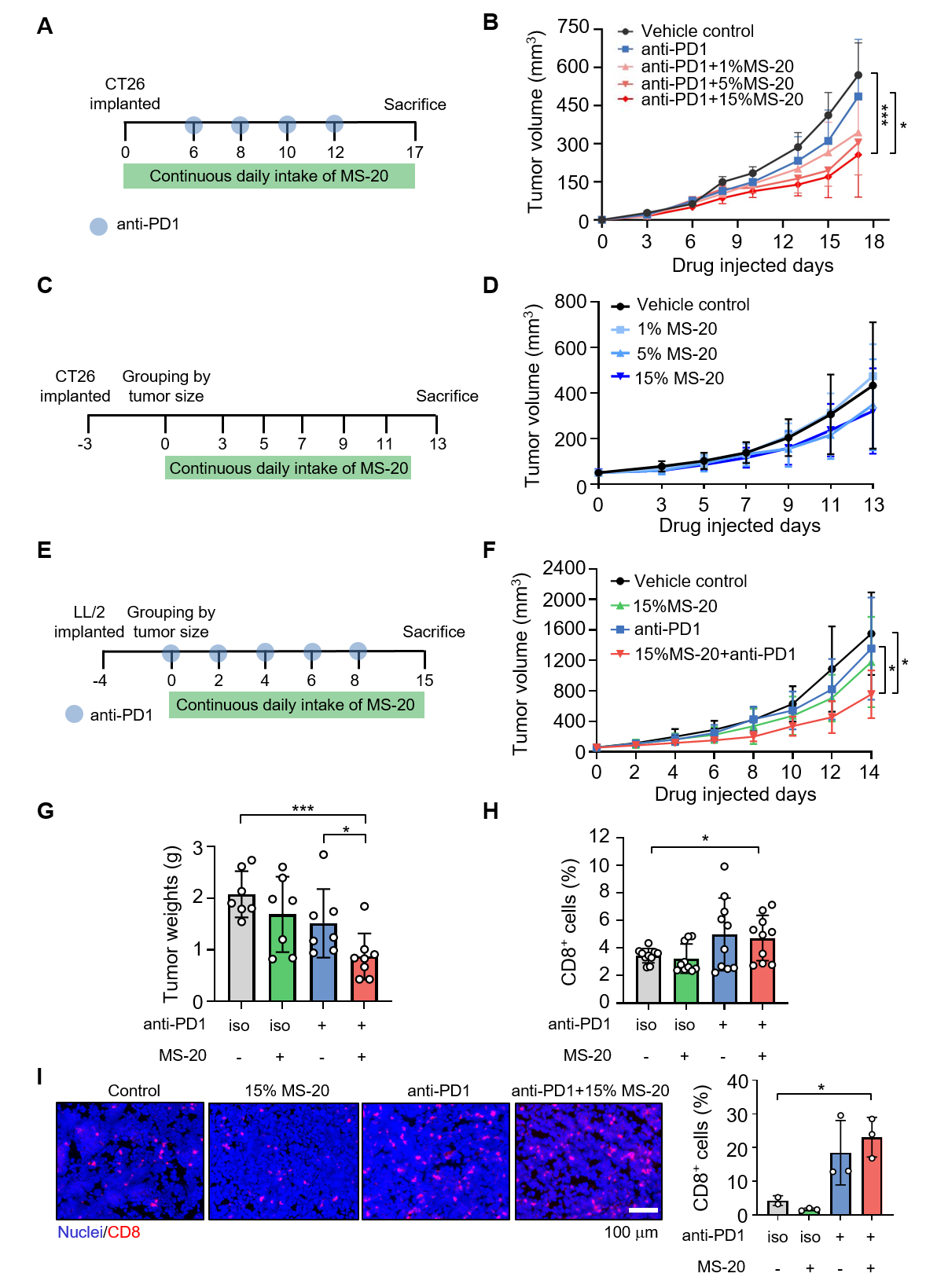


**Figure S1 MS-20 in combination with an anti-PD1 antibody inhibited tumor growth in a colon and lung cancer xenograft model.**

A prevention model was used to study the antitumor activity of MS-20 in combination with anti-PD1 therapy. After CT-26 cancer cells implantation, mice were subsequently randomized into the vehicle control, anti-PD1 alone or in combination with 1%, 5% or 15% MS-20 orally daily groups. An anti-PD1 antibody was intraperitoneally injected at the indicated time points (A). Tumor growth in the prevention model was monitored (n=6/group) (B). To examine the effects of MS-20 alone on tumor growth, CT-26 cancer cells were implanted and mice were randomly grouped into vehicle control or 1%, 5% or 15% MS-20 orally daily after tumor growth (C). Tumor growth of different doses of MS-20 was monitored (n=12/group) (D). A total of 10^6^ viable LL/2 cells were subcutaneously injected into C57BL/6 mice. After tumor growth, the mice were randomly assigned to the control, 15% MS-20, anti-PD1 antibody or combination therapy groups (E). Tumor growth of the LL/2 xenograft model was measured (n=7~8/group) (F). Tumor weights of LL/2 in the control, 15% MS-20, anti-PD1 and 15% MS-20 plus anti-PD1 groups were determined (n=7~8/group) (G). Tumor-infiltrating CD8^+^ T cells were determined using IHC staining. The number of cells in whole tumor tissue was quantified using HALO software, and the percentage of CD8^+^ cells was calculated as the sum of CD8^+^ cells divided by the total number of nuclear cells (n=10/group) (H). Immunofluorescence images were collected and intratumoral CD8^+^ cells was determined (n=3/group) (I). The data represent the mean±sd (B, D, F, G, H, I). The differences were assessed using Student’s t test B, D, F, G, H, I). *p≤0.05, **p≤0.01 and ***p≤0.001. iso: isotype control.


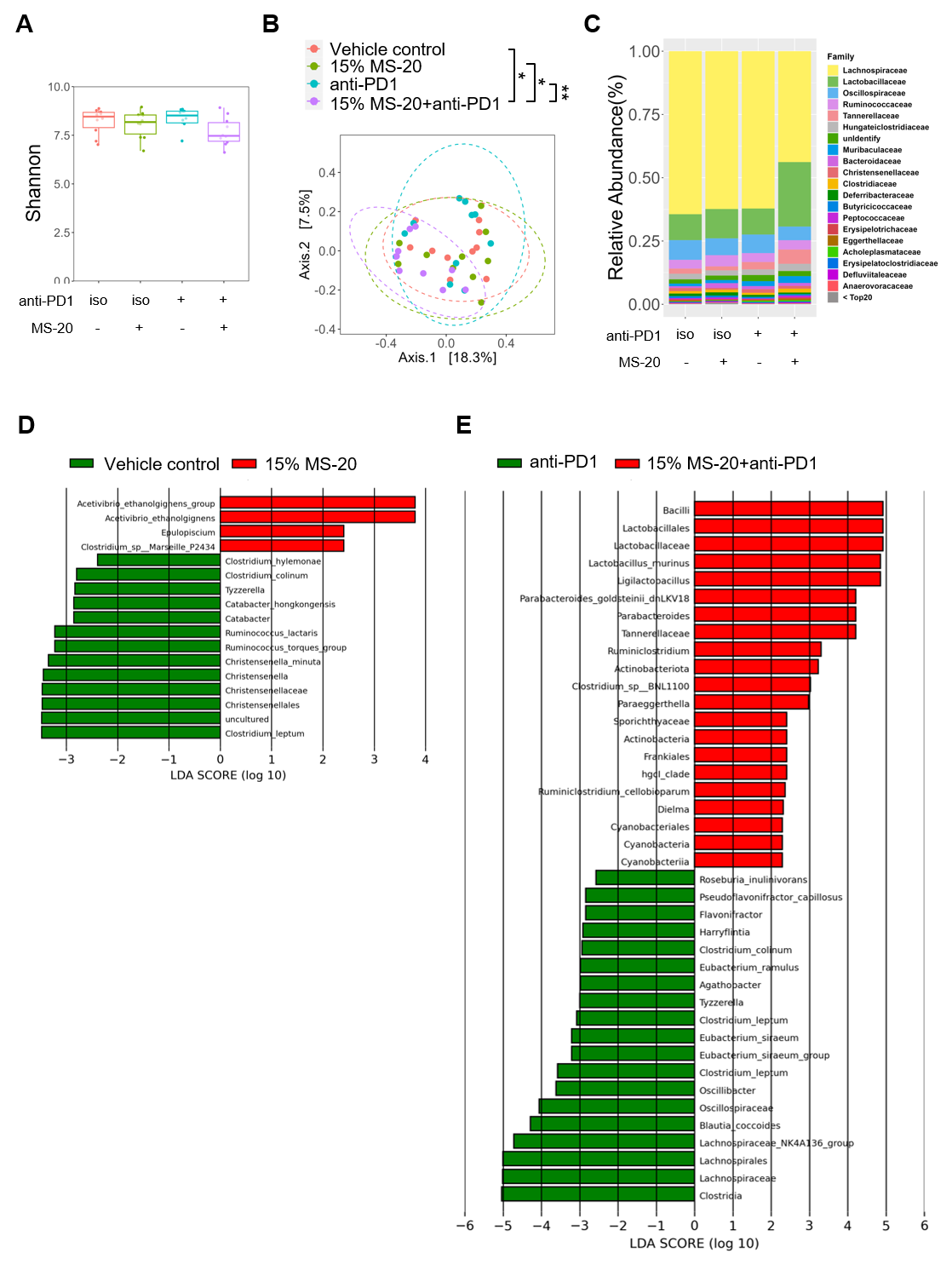


**Figure S2 Analysis of gut microbiota composition following treatment with MS-20 in combination with an anti-PD1 antibody.**

A 16S RNA sequence analysis was performed to assess the α-diversity (A) and β-diversity (B) of the vehicle control, 15% MS-20, anti-PD1 and 15% MS-20 in combination with anti-PD1 groups. Bacterial composition of the four groups at the family level was assessed (C). LEfSe analysis revealed differential bacterial expression between the vehicle control group and 15% MS-20 group (D) and the15% MS-20 combined with anti-PD1 therapy versus anti-PD1 therapy alone groups (E). Bacteria with LDA score ≥ 2 were shown. The differences were assessed using Student’s t test (A) and Permutation testing (B). *p≤0.05, **p≤0.01 and ***p≤0.001. iso: isotype control.


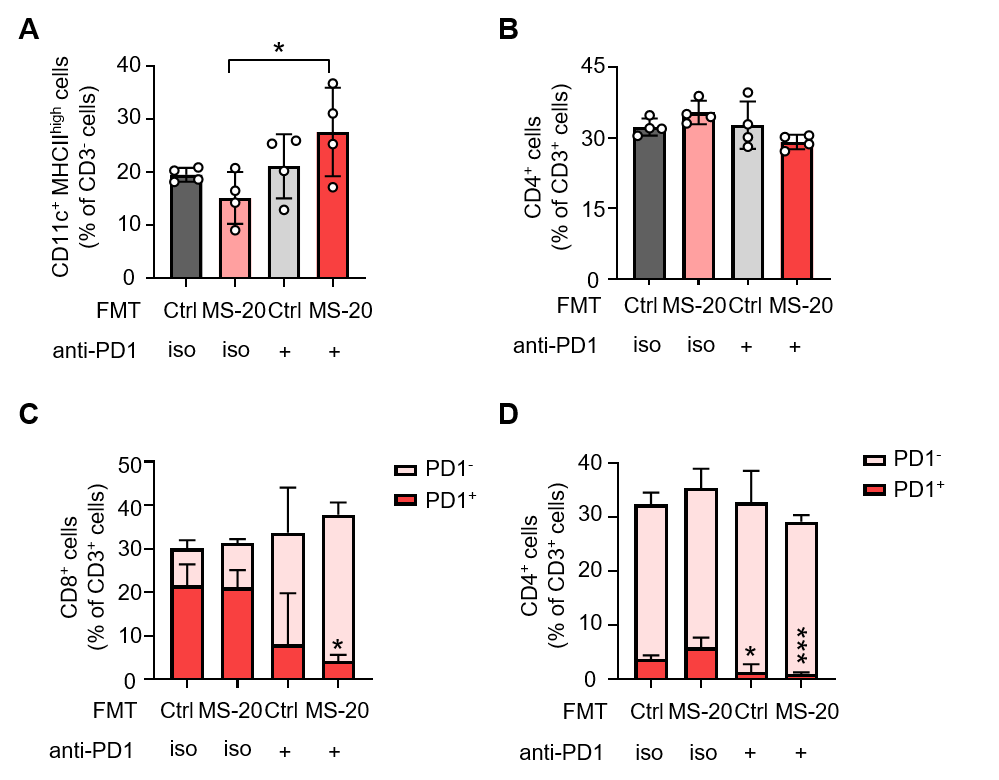


**Figure S3 Tumor-infiltrating immune cells after MS-20 fecal plus anti-PD1 antibody treatment.**

Flow cytometry analysis was performed to analyze CD11c^+^ MHCII^high^ cells (n=4/group) (A) CD4^+^ T cells (n=4/group) (B) CD8^+^PD1^+^ and CD8^+^PD1^-^ T cells (n=4/group) (C) and CD4^+^PD1^+^ and CD4^+^PD1^-^ T cells (n=4/group) (D) in the tumor microenvironment. The data represent the mean±sd. The differences were assessed using Student’s t test. *p≤0.05, **p≤0.01 and ***p≤0.001. iso: isotype control.


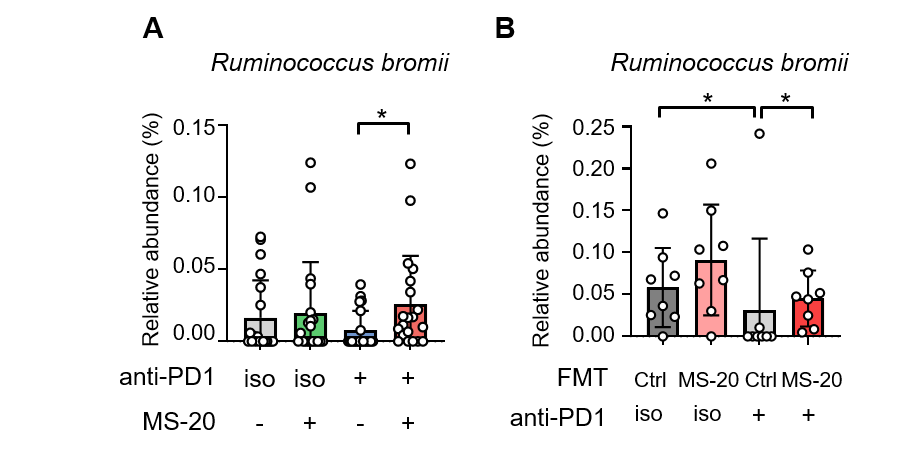


**Figure S4 Relative abundance of *R. bromii* in the animal model.**

The relative abundance of *R. bromii* in mice in the vehicle control, 15% MS-20, anti-PD1 and 15% MS-20 plus anti-PD1 groups was determined (n=20/group) (A). The relative abundance of *R. bromii* in mice that received vehicle control or MS-20-treated feces with or without cotreatment with anti-PD1 was determined (n=8/group) (B). The data represent the mean±sd. The differences were assessed using Student’s t test. *p≤0.05, **p≤0.01 and ***p≤0.001.


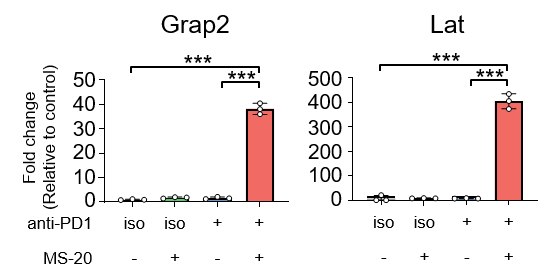


**Figure S5 Expression of genes related to TCR signaling.**

The gene expression levels of Grap2 and Lat in colon epithelial cells from the vehicle control, 15% MS-20, anti-PD1 and combination groups. The data represent the mean±sd. The differences were assessed using Student’s t test. N=3/group. *p≤0.05, **p≤0.01 and ***p≤0.001.


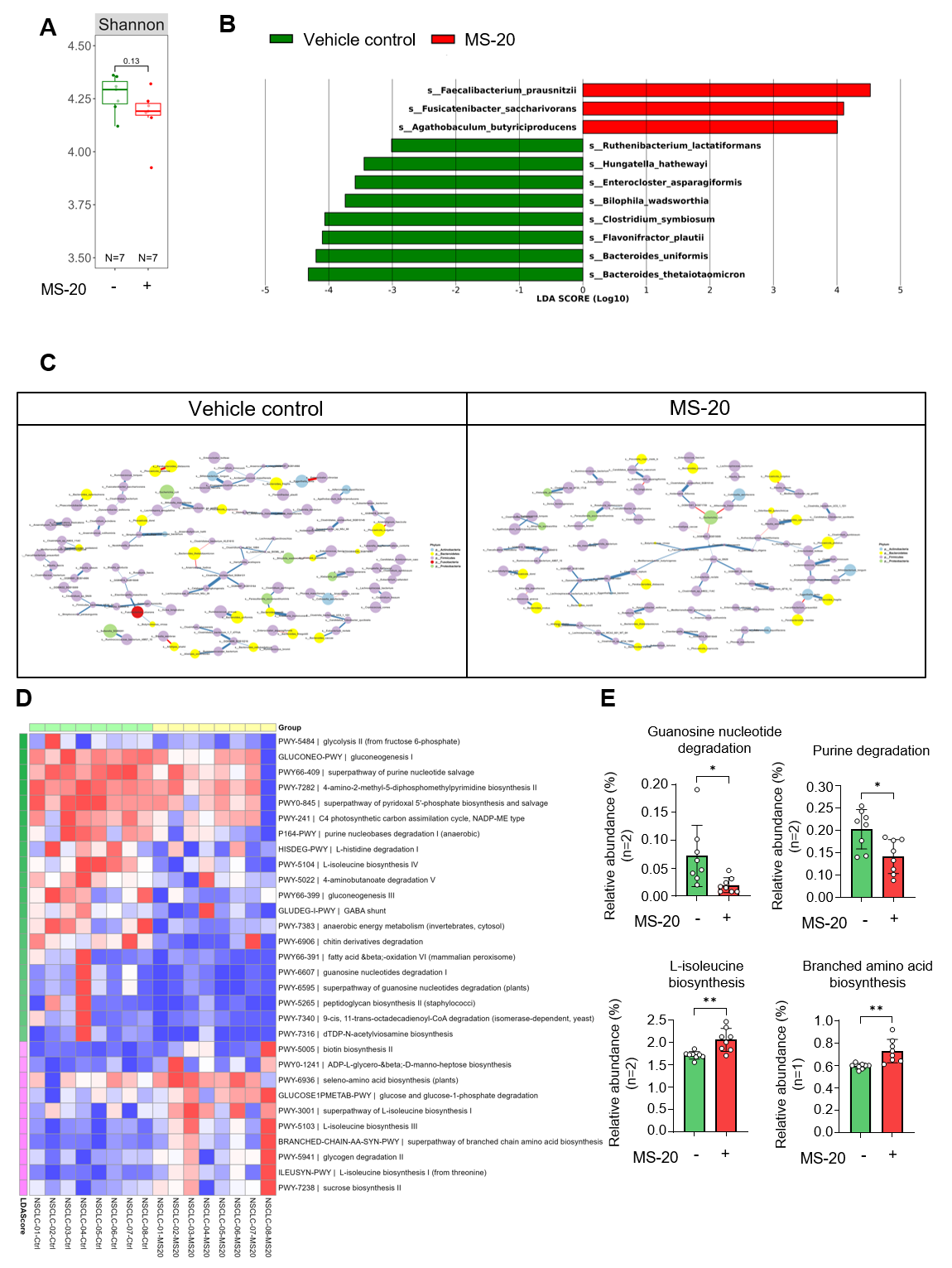


**Figure S6 Metagenomic analysis revealed that MS-20 regulated the microbiota of cancer patients.**

The α-diversity of CRC fecal samples after vehicle control treatment and 2.5% MS-20 treatment was determined (A). LEfSe was used to identify differentially abundant bacteria of NSCLC fecal samples treated with vehicle control and 2.5% MS-20 (B). The co-occurrence networks were generated to compare the vehicle control and MS-20 treatment groups of NSCLC fecal samples (C). NSCLC fecal samples treated with or without MS-20 were analyzed by MetaCyc to predict metabolic pathway enrichment. Pathways related to differentially abundant genes in the control group versus MS-20 group were analyzed by LEfSe. A heatmap was generated to show the abundance of each outcome-associated pathway in each sample; the largest value was defined as the maximum, and the smallest value was defined as the minimum (D). The relative abundance of significant metabolic pathways that were classified in the same category according to MetaCyc were added together and are shown for individual samples. Each dot represented an individual sample. (n=8/group) (E). The data represent the mean±sd. The differences were assessed using the Student’s t test (A, E). *p≤0.05, **p≤0.01 and ***p≤0.001.
